# Thermal Changes During Clavicle Fracture Healing in Children

**DOI:** 10.3390/jcm13237213

**Published:** 2024-11-27

**Authors:** Filip Jurić, Anko Antabak, Ivonne Žgaljardić, Ana Bosak Veršić, Suzana Sršen Medančić, Goran Augustin

**Affiliations:** 1 Department of Pediatric Surgery, Clinical Hospital Center Rijeka, 51000 Rijeka, Croatia; filip.juric@uniri.hr (F.J.); ana.bosak.versic@medri.uniri.hr (A.B.V.); suzanasm@medri.uniri.hr (S.S.M.); 2 School of Medicine, University of Zagreb, Šalata 3, 10000 Zagreb, Croatia; aantabak@kbc-zagreb.hr (A.A.); zgaljardic.ivonne@gmail.com (I.Ž.); 3 Department of Pediatric Surgery, University Hospital Centre Zagreb, Kišpatićeva 12, 10000 Zagreb, Croatia; 4 Aesthetic Surgery Center dr. Žgaljardić, Nova cesta 46B, 51210 Opatija, Croatia; 5 Department of Surgery, University Hospital Centre Zagreb, Kišpatićeva 12, 10000 Zagreb, Croatia

**Keywords:** clavicle fractures, fractures, bone, upper extremity, child, adolescent, radiography, infrared thermography, callus, pediatric surgery, pediatric orthopedics

## Abstract

**Introduction:** Clavicle fractures are among the most common in children, typically treated conservatively, with standard radiographs used to diagnose and monitor healing. Recently, infrared thermography (IRT) has been proposed as an alternative method for fracture detection, but no study has correlated the temperature changes during callus formation. **Materials and Methods:** Children aged 4–18 with X-ray-diagnosed clavicle fractures were included in the study. IRT measured temperatures above the fracture and contralateral healthy side on the 1st, 4th, 8th, 15th, and 22nd day after the injury. Along with IRT, an ultrasound was used to assess callus formation. **Results:** The study included 27 patients with an average age of 12.4 years, mostly boys. The left side was more often affected than the right side (33%). We found a correlation between callus formation and the ∆T. A maximum temperature difference of an average of 0.7 °C was noted during the proliferative phase of callus formation. After the formation of the fibrocartilaginous callus (4th to 8th day), the temperature above the fracture declined until it was equal (22nd day) to that of the healthy side. The average temperature difference between the broken and the healthy sides was statistically significant on the 4th and 8th days (during callus formation). **Conclusions:** The increased skin temperature above the fracture correlates with the inflammatory phase of bone healing. After the callus is visible on ultrasound, the temperature linearly drops with no statistical difference between the injured and the healthy sides. The standard protocol for clavicle fracture treatment typically involves using X-rays to assess callus formation during follow-up. IRT has shown potential in diagnosing callus formation in children with clavicle fractures, potentially reducing the need for traditional X-rays.

## 1. Introduction

Clavicle fractures are one of the most prevalent fractures in children, constituting 8–15% [1,2] of upper limb fractures. Children across various age groups are susceptible to these fractures, with injury mechanisms differing based on the child’s age. Younger children primarily sustain injuries from falls at heights, whereas school-age children and adolescents often obtain fractures due to falls during sports activities (such as football) or while cycling [2,3]. The midshaft region of the clavicle is most frequently affected, accounting for approximately 80% of cases. Notably, a substantial proportion (approximately 64%) of these fractures exhibit minor or no fragment displacement [4].

In most childhood cases, clavicle fractures are treated conservatively, reserving surgical intervention for specific indications. Radiographic imaging plays a pivotal role in monitoring fragment alignment and clavicle healing, indicated by the appearance of a solid periosteal callus. A three-week follow-up period is typically observed, with immobilization removed once a robust bony callus has been formed.

The blood flow influences skin temperature the most. Bone healing is a complex process with three phases: inflammatory, proliferative, and remodeling. The inflammatory phase (angiogenesis phase) lasts 4–7 days and is characterized by the organization of hematoma, angiogenesis, and the formation of inflammatory granulation tissue rich in newly formed capillaries. The proliferative phase is marked by the proliferation of osteogenic cells, mainly derived from the periosteum, which forms the external callus. During this phase, a fibrocartilaginous callus is formed. Within 5–11 days, callus can be verified by X-rays in children. The next phase involves cellular organization, where the surrounding fibrous tissue serves as a scaffold over which cells migrate to stabilize the fracture. Mesenchymal cells attracted by various growth factors differentiate into osteoblasts and chondroblasts, creating new bone. This healing phase lasts 3–4 weeks. The final phase of bone healing is the remodeling phase, characterized by the resorption of excessive and unnecessary portions of the callus and the orientation of trabecular bone along the lines of the stress on the bone. This final phase of bone healing can last several years.

Infrared thermography (IRT) detects energy from the infrared spectrum, which is invisible to the human eye. The program implemented in the camera’s software translates this spectrum into the visible spectrum by assigning a specific color shade to each temperature, where blue represents the lowest and white the highest temperature [5]. With the advancement of technology, thermal cameras have become sufficiently accurate and sensitive, and the use of IRT in medicine has increased in recent years. In cancer patients with breast carcinoma, IRT has been accepted as one of the diagnostic methods [6,7,8,9]. In addition to diagnosing breast cancer, there are increasingly promising studies on detecting malignant changes in other organs, such as the thyroid [9,10]. Beyond oncology, IRT is routinely used for detecting Raynaud’s syndrome [9,11,12]. Some recent research focuses on the usefulness of thermography in locating perforators and assessing the vitality of flaps in plastic-reconstructive surgery [9,13,14,15]. Moreover, IRT shows promising results as a valuable tool for diagnosing appendicitis, monitoring skin diseases, and aiding in the diagnosis of autism (facial mimic), type II diabetes (periorbital region), and various eye diseases [9].

Recently, IRT has been proposed as an alternative method for fracture detection. Most studies have focused on detecting fractures of the upper extremities [16,17,18]. Children represent a specific population where new fracture detection methods are sought due to the negative effects of X-ray radiation. Hence, it is unsurprising that most studies are directed toward using IRT as a screening method for identifying fractures in children [17,18,19]. Sanchis-Sánchez et al. emphasize that IRT is a promising method for fracture detection with a sensitivity of 91%, specificity of 88%, and predictive value of 95%, noting that lesion size is crucial for the discriminatory power of the method [19]. Ćurković et al. observed a temperature difference in pediatric radius fractures ranging from 0.8 to 2 °C compared to the healthy side, with this value decreasing as the fracture heals [18].

This study aims to determine the difference and dynamics of temperature changes above the fractured clavicle compared to the healthy side and observe how the dynamics of temperature changes correspond to the formation of callus.

## 2. Materials and Methods

This is a prospective study involving children aged 4–18 who presented to the surgical emergency department with a clavicle fracture. The diagnosis was made based on clinical examination and X-ray imaging. Exclusion criteria for this study include subperiosteal fractures, patients requiring surgical treatment, patients with skin infections or skin diseases, and patients with rheumatoid arthritis or diabetes. Written informed consent was obtained from the patients and their guardians. The hospital’s ethics board committee granted ethical approval for this study.

The TrueIR Thermal Imager Model U5855A, manufactured by Keysight Technologies (Santa Rosa, CA, USA), was utilized for thermographic measurements. The sensitivity of the camera is 0.07 °C at an ambient temperature of 30 °C. The thermal imager (TI) generated graphical maps, or thermograms, which were subsequently processed using the manufacturer’s original software, “TrueIR Analysis and Reporting Tool” version 2.0.60920.01 (Keysight Technologies, Santa Rosa, CA, USA). To allow the camera to warm up and thus obtain the most accurate data (recommended by the manufacturer), the camera was turned on at least 5 min before capturing the thermogram.

All patients were treated conservatively. An X-ray was used for the initial diagnosis of the fracture and later during follow-up only if indicated by the attending physician. All patients were treated according to the standard protocol for clavicle fracture management in children, which included wearing a sling for three weeks.

**Figure 1:**
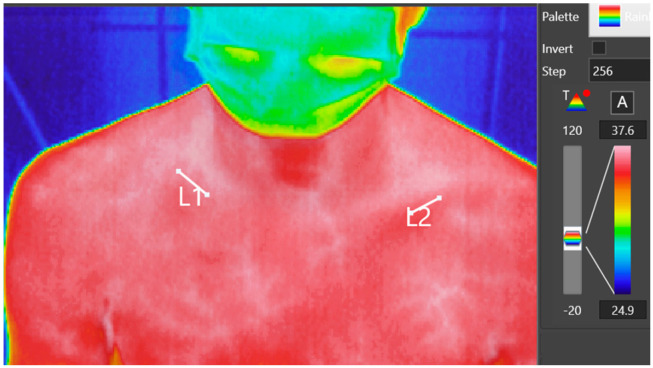
Thermogram in Keysight TrueIR Analysis software with the selected area of fracture (L1) and same place on the healthy side (L2). The patient wears a mask (COVID-19 pandemic time).

A standardized protocol was implemented for all patients, ensuring consistency in environmental and imaging conditions. This protocol included maintaining a stable room temperature of 22–24 °C and a humidity level of approximately 50%. The thermal imaging camera was positioned 1 m from each subject to maintain uniformity in image resolution and accuracy across all examinations. Prior to imaging, the sling worn by subjects was removed 10 min in advance to minimize any residual thermal effects from the fabric on the skin’s surface temperature. After the examination, the sling was carefully re-positioned to maintain patient comfort and adherence to treatment protocols. During imaging, the thermal camera captured both clavicles within a single thermogram, encompassing the relevant portion of the torso. This setup allowed for direct comparison of thermal data between the injured and non-injured clavicle. On the thermogram, markings were placed over the clinically identified fracture area and the corresponding spot on the healthy side. The average temperature of these marked areas was then measured using the manufacturer’s software (Figure 1).

**Figure 2:**
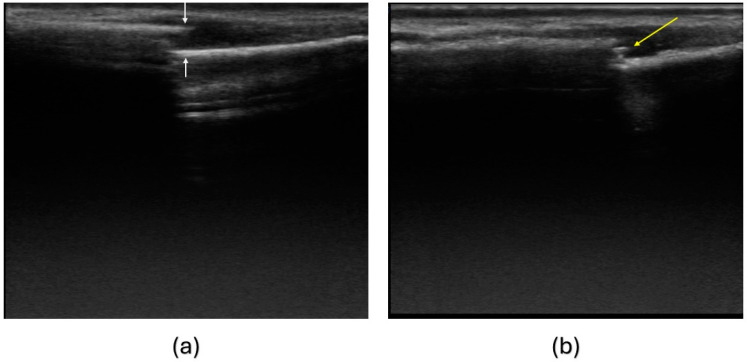
Ultrasound examination of a patient. (**a**) The ultrasound image on the 4th day after the injury showed no visible callus. The white arrows point to fracture ends. (**b**) Ultrasound image on the 8th post-injury day, with the yellow arrow pointing to the callus formation.

The initial measurement was conducted within 24 h of the injury. Subsequent appointments were scheduled on days 4, 8, 15, and 22. Following IRT, ultrasound imaging of the fractured bone was performed as part of the examination to assess callus formation. The ultrasound examination was performed by the radiology specialist (Figure 2).

Statistical analysis was carried out using an ANOVA for repeated measures, and data processing was conducted utilizing MedCalc software, version 20.104 (MedCalc Software Ltd., Ostend, Belgium).

## 3. Results

A total of 27 children were included, with an average age of 12.4 (range 6–17). Most were boys (22 patients). The left side exhibited a higher occurrence rate (18 vs. 9).

**Figure 3:**
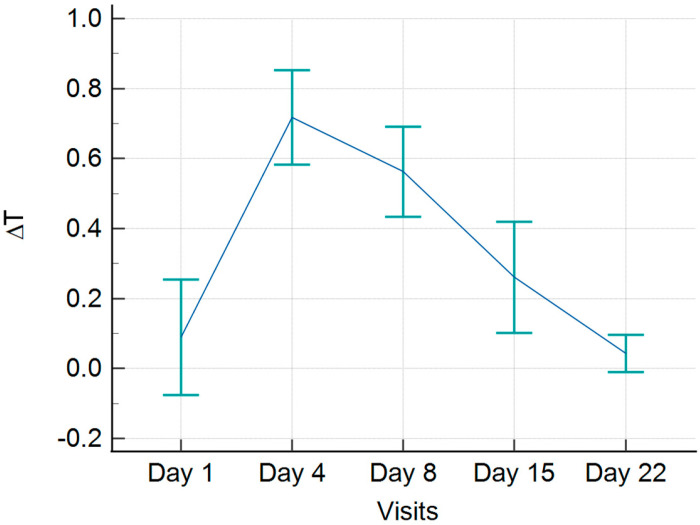
Temperature difference (∆T) in °C during clavicle healing between injured and healthy side.

We found that the skin temperature increased above the affected side during the proliferative phase of callus formation, with an average value of 0.7 °C. After reaching a peak on the 4th day, the curve slightly declined until the 8th day with an average ∆T of 0.6 °C, followed by a linear decrease until the 22nd day, when no difference was observed between the healthy and injured sides (Figure 3).

A repeated measures ANOVA revealed a significant main effect of the factor, F(4,104) = 26.65, *p* < 0.001. When comparing the average temperature difference between the fractured and the healthy side across time points, a statistically significant difference is evident between 4th and 8th day in contrast to the other visits. Specifically, the *p*-values for the temperature difference between the 4th day and the 1st, 15th, and 22nd days are *p* < 0.0001, *p* < 0.0003, and *p* < 0.0001, respectively. Similarly, the *p*-values for the temperature difference between the 8th day and the 1st, 15th, and 22nd days are *p* = 0.0001, *p* = 0.0020, and *p* < 0.0001. Importantly, no statistically significant differences were observed between the temperature readings on the 4th and the 8th day following the injury (Table 1).

**Figure 4:**
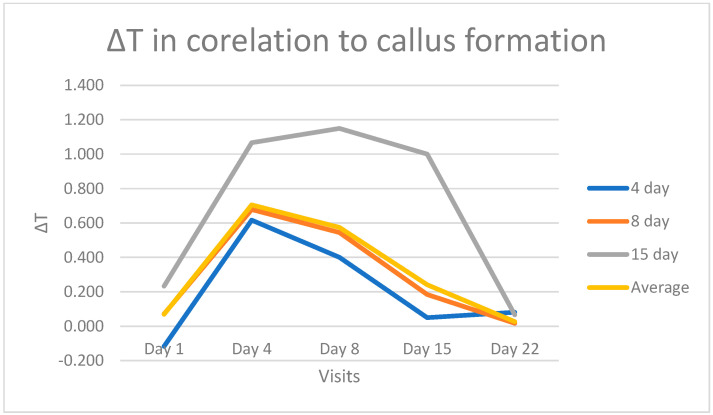
Temperature difference in °C (∆T) in correlation to callus formation.

For most patients, callus formation became evident and detectable by ultrasound on the 8th day post-injury. In two cases, callus was visibly formed by the 4th day, while in another two cases, this was observed on the 15th day following injury. A noteworthy observation is that when comparing the temperature curves of these patients, those who exhibited callus formation on the 4th day displayed a sharper decline in the curve, with equalization observed by day 15. In contrast, patients who developed a callus on the 15th day presented a higher ∆T with a maximum of 1.2 °C on the 8th day, followed by a decrease until normalization on the 22nd day (Figure 4).

**Figure 5:**
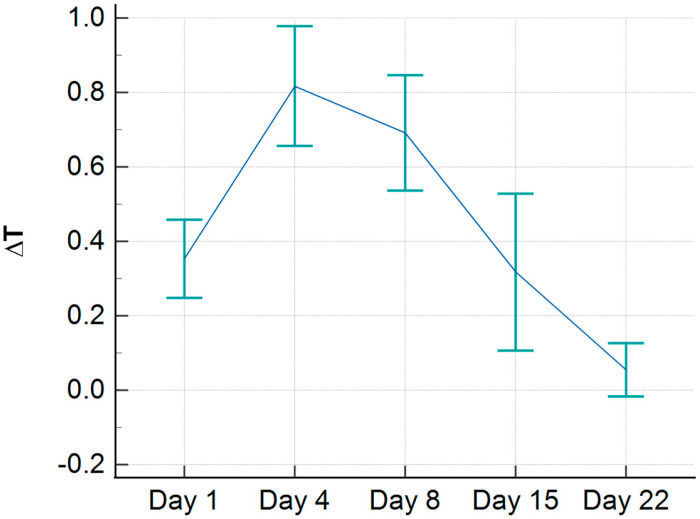
The graph represents temperature difference (∆T) between injured and healthy side after excluding patients who had a negative ∆T during the initial assessment. It shows that ∆T at the initial assessment is now elevated, with an average value of 0.35 °C. The peak value, which is on the 4th day, is also elevated with a value of 0.82 °C.

During the study, we observed that a certain number of children (10 in total) presented with a negative ∆T during the initial assessment, meaning that the injured side was colder than the healthy side. When these patients were excluded, the average temperature during the initial assessment was elevated compared to the healthy side by 0.35 °C (Figure 5). Furthermore, the peak temperature difference, observed on the 4th day, was also slightly higher, with a value of 0.82 °C.

If we statistically compare the temperature differences after the patients with negative ∆T were excluded (Table 2), we obtain similar results as when all study participants are included. A repeated measures ANOVA revealed a significant main effect of the factor F(4,64) = 22.13, *p* < 0.001. Notably, a statistically significant difference now emerges between the initial and final follow-up measurements (*p* < 0.05). The ∆T values on the 4th and 8th post-injury days still differed significantly from other examination time points, with *p* < 0.05 or even *p* < 0.0001 when compared to the 22nd day.

## 4. Discussion

The findings of this study align with the previous research, reinforcing the established link between an increase in skin temperature above the fracture site [16,17,18,19]. In our study, the highest average difference observed between the fractured and the healthy side was 0.7 °C. Most patients reached maximum temperature difference on the 4th day following trauma, after which the temperature gradually decreased until the 8th day. This pattern closely corresponds to the inflammatory phase of bone healing, which typically lasts approximately one week [20]. The initiation of fibrocartilaginous callus formation usually occurs between days 5 and 11 [20], which concurs with the observed minor temperature decline in our study.

Furthermore, our study found that most patients developed fibrocartilaginous callus by the 8th day, as confirmed by ultrasound. After the formation of the fibrocartilaginous callus and the progression to bony callus formation, typically between days 11 and 21 [20], the temperature dropped, ultimately normalizing and becoming equivalent to the healthy side.

Remarkably, some patients displayed visible fibrocartilaginous callus formation on the 4th day, while others showed the same changes on the 15th day, confirmed with ultrasound. The temperature curves in these patients closely mirrored the timing of callus formation. In patients with callus formation on the 4th day, the temperature curve was narrower and steeper, with temperature differences equalizing by the 15th day. Conversely, in patients with callus formation on the 15th day, the curve exhibited a wider peak temperature on the 8th day, but regardless, it eventually declined and normalized by the 21st day (Figure 4). These observations underscore the dynamic relationship between callus formation and temperature changes during healing. However, the sample size (only two patients) is too small to provide statistically significant evidence.

When comparing temperature differences between visits, particularly between the 4th and 8th days and other visits, a statistically significant difference is observed compared to the temperature readings from the 1st, 15th, and 22nd days (Table 1). It is worth highlighting that there was no statistically significant difference when comparing the temperature readings between the 4th and 8th day. These findings align with the previously discussed phases of bone healing, indicating a significant rise in temperature during the inflammatory phase and the formation of the fibrocartilaginous callus, which, together, extend for up to 11 days. Conversely, the 4th and 5th visits correspond to the period when bony callus formation predominates; this phase extends up to 21 days.

Previous publications suggest that IRT is a safe and reliable method for detecting bone fractures, demonstrating high sensitivity and specificity [19]. However, in our findings, we did not observe a statistically significant increase in temperature within the first 24 h post-injury. Sanchis-Sánchez, in his publication, excluded 12 patients because the temperature on the injured side was lower than on the healthy side. Similarly, in our research, we identified 10 patients with comparable findings. When we excluded these patients from our study, we found that the temperature difference at the time of injury (within the first 24 h) was elevated by an average of 0.35 °C, which was statistically significant compared to the final visit on the 22nd day. The peak temperature difference reached 0.82 °C; however, the temperature gradually decreased and equalized by the 22nd day. We hypothesize that the hematoma formed after the injury may act as a barrier, impeding heat transfer to the skin. Further research is needed to investigate this phenomenon in greater detail.

Our findings have revealed a correlation between temperature changes and bone healing phases, highlighting the potential utility of IRT as a valuable tool for monitoring stages of fracture healing, particularly clavicle fractures.

This development is significant, especially in the pediatric population, where minimizing the use of harmful X-ray radiation is a paramount concern. While X-rays have traditionally been the gold standard for monitoring fracture healing, our study suggests that IRT could offer a less invasive and radiation-free alternative. According to our findings, when the temperature over the fractured clavicle aligns with that of the normal side, it signifies the formation of a bony callus, suggesting that the fracture has stabilized sufficiently to allow for the safe removal of immobilization. The current routine for monitoring clavicle fractures often involves the use of X-rays to confirm callus formation.

IRT offers immediate and non-contact assessment, making it particularly useful in scenarios where access to radiographic facilities is limited or unavailable. For example, this approach could be valuable in underdeveloped countries or remote settings, such as displaced ambulance units without access to X-ray equipment.

As evidenced by numerous studies, ultrasound is another non-invasive method that can be used to monitor fractures. In the systematic review, Ackermann reported that the sensitivity of ultrasound in detecting clavicle fractures is 91%, while its specificity is 93% [21]. However, the drawbacks of ultrasound as a diagnostic tool include the relatively long examination time (up to 15 min per patient), especially in small, uncooperative children, moderate discomfort during the procedure, and the requirement for trained personnel to perform the examination. Additionally, the curved shape of the clavicle can sometimes make the examination challenging to perform. In contrast, infrared thermography (IRT), with comparable sensitivity and specificity (91% and 88%, respectively), offers a diagnostic method that is quick and painless. With advancements in artificial intelligence and technology, it is conceivable that IRT could evolve into a tool requiring minimal or no user training, making it more accessible for widespread use.

Future studies should aim to establish standardized IRT protocols, define baseline temperature patterns for various types of fractures, and validate these findings in larger and more diverse patient populations. Additionally, research should explore temperature patterns associated with pathological conditions like pseudarthrosis and malunion to assess the potential of IRT in detecting and monitoring these complications. This promising avenue of research could pave the way for the broader application of IRT in orthopedics, potentially revolutionizing how we monitor and manage fracture healing in various clinical settings.

We believe even better results could be achieved with IRT with higher resolution. The resolution of our camera was only 320 × 240 pixels. While collecting data, we had to be very precise. An IRT with higher resolution can capture finer temperature variations and provide more precise data. This increased level of detail can be especially advantageous when monitoring subtle temperature changes associated with the different phases of bone healing. With a higher-resolution IRT, these meticulous measurements can become even more effective, enhancing reliability and specificity. Another limitation of this study is the small number of patients included. We only had 2 patients who showed callus formation on the 4th day and 2 patients who had formed callus by the 15th day. While their curves follow the expected pattern of callus formation, further investigation is needed. Additionally, the study was limited to 10 patients with negative ∆T values, which influenced the average temperature measurements during the first 24 h post-injury.

## 5. Conclusions

This study confirms the findings of earlier research that utilized IRT to identify temperature increases at the site of bone fractures. Furthermore, it also reveals an association between temperature changes and callus formation. Temperature is elevated during the inflammation phase and callus formation. Once the callus forms, the temperature drops and equalizes with the healthy side. Further investigations are needed to determine the temperature curve for other fractures. While traditionally, X-rays are used to monitor the healing process of a fractured bone, IRT could offer a less invasive, radiation-free alternative. This is especially important in the pediatric population.

## Figures and Tables

**Table 1:** Statistical correlation of temperature difference between visits.

Relation of Visits			Mean Difference	Std. Error	*p*	95% CI
Day_1	–	Day_4	−0.629	0.0820	<0.0001	−0.880 to −0.378
	–	Day_8	−0.474	0.0802	<0.0001	−0.720 to −0.228
	–	Day_15	−0.172	0.105	1.0000	−0.495 to 0.151
	–	Day_22	0.0460	0.0873	1.0000	−0.222 to 0.314
Day_4	–	Day_1	0.629	0.0820	<0.0001	0.378 to 0.880
	–	Day_8	0.155	0.0668	0.2822	−0.0496 to 0.360
	–	Day_15	0.457	0.0900	0.0003	0.181 to 0.733
	–	Day_22	0.675	0.0789	<0.0001	0.433 to 0.917
Day_8	–	Day_1	0.474	0.0802	<0.0001	0.228 to 0.720
	–	Day_4	−0.155	0.0668	0.2822	−0.360 to 0.0496
	–	Day_15	0.302	0.0697	0.0020	0.0881 to 0.516
	–	Day_22	0.520	0.0632	<0.0001	0.326 to 0.714
Day_15	–	Day_1	0.172	0.105	1.0000	−0.151 to 0.495
	–	Day_4	−0.457	0.0900	0.0003	−0.733 to −0.181
	–	Day_8	−0.302	0.0697	0.0020	−0.516 to −0.0881
	–	Day_22	0.218	0.0783	0.0986	−0.0221 to 0.458
Day_22	–	Day_1	−0.0460	0.0873	1.0000	−0.314 to 0.222
	–	Day_4	−0.675	0.0789	<0.0001	−0.917 to −0.433
	–	Day_8	−0.520	0.0632	<0.0001	−0.714 to −0.326
	–	Day_15	−0.218	0.0783	0.0986	−0.458 to 0.0221

**Table 2:** Statistical correlation of temperature difference between visits after the patients with negative ∆T were excluded.

Relation of Visits			Mean Difference	Std. Error	*p*	95% CI
Day_1	–	Day_4	−0.464	0.0906	0.0010	−0.759 to −0.170
	–	Day_8	−0.339	0.0854	0.0110	−0.617 to −0.0612
	–	Day_15	0.0346	0.110	1.0000	−0.323 to 0.393
	–	Day_22	0.298	0.0697	0.0057	0.0719 to 0.525
Day_4	–	Day_1	0.464	0.0906	0.0010	0.170 to 0.759
	–	Day_8	0.125	0.0842	1.0000	−0.149 to 0.399
	–	Day_15	0.499	0.109	0.0031	0.145 to 0.853
	–	Day_22	0.763	0.0942	<0.0001	0.456 to 1.069
Day_8	–	Day_1	0.339	0.0854	0.0110	0.0612 to 0.617
	–	Day_4	−0.125	0.0842	1.0000	−0.399 to 0.149
	–	Day_15	0.374	0.0897	0.0073	0.0817 to 0.665
	–	Day_22	0.637	0.0770	<0.0001	0.387 to 0.888
Day_15	–	Day_1	−0.0346	0.110	1.0000	−0.393 to 0.323
	–	Day_4	−0.499	0.109	0.0031	−0.853 to −0.145
	–	Day_8	−0.374	0.0897	0.0073	−0.665 to −0.0817
	–	Day_22	0.264	0.104	0.2188	−0.0740 to 0.602
Day_22	–	Day_1	−0.0346	0.0697	0.0057	−0.525 to −0.0719
	–	Day_4	−0.499	0.0942	<0.0001	−1.069 to −0.456
	–	Day_8	−0.374	0.0770	<0.0001	−0.888 to −0.387
	–	Day_15	0.264	0.104	0.2188	−0.602 to 0.0740

## Data Availability

The data that support the findings of this study are available from the corresponding author upon reasonable request. Before this request, users should obtain permission from the local ethics committee.
